# Identification of metabolic fingerprints in severe obstructive sleep apnea using gas chromatography–Mass spectrometry

**DOI:** 10.3389/fmolb.2022.1026848

**Published:** 2022-11-21

**Authors:** Mohit, Manendra Singh Tomar, Fabrizio Araniti, Ankit Pateriya, Ram Awadh Singh Kushwaha, Bhanu Pratap Singh, Sunit Kumar Jurel, Raghuwar Dayal Singh, Ashutosh Shrivastava, Pooran Chand

**Affiliations:** ^1^ Department of Prosthodontics, Faculty of Dental Sciences, King George’s Medical University, Lucknow, India; ^2^ Center for Advance Research, Faculty of Medicine, King George’s Medical University, Lucknow, India; ^3^ Dipartimento di Scienze Agrarie e Ambientali, Produzione, Territorio, Agroenergia (DiSAA), University of Milan, Milan, Italy; ^4^ Department of Respiratory Medicine, Faculty of Medicine, King George’s Medical University, Lucknow, India; ^5^ Midland HealthCare and Research Centre, Lucknow, India

**Keywords:** obstructive sleep apnea, metabolomics, gas chromatography–mass spectrometry, biomarkers, polysomnography

## Abstract

**Objective:** Obstructive sleep apnea (OSA) is considered a major sleep-related breathing problem with an increasing prevalence rate. Retrospective studies have revealed the risk of various comorbidities associated with increased severity of OSA. This study aims to identify novel metabolic biomarkers associated with severe OSA.

**Methods:** In total, 50 cases of OSA patients (49.74 ± 11.87 years) and 30 controls (39.20 ± 3.29 years) were included in the study. According to the polysomnography reports and questionnaire-based assessment, only patients with an apnea–hypopnea index (AHI >30 events/hour) exceeding the threshold representing severe OSA patients were considered for metabolite analysis. Plasma metabolites were analyzed using gas chromatography–mass spectrometry (GC-MS).

**Results:** A total of 92 metabolites were identified in the OSA group compared with the control group after metabolic profiling. Metabolites and their correlated metabolic pathways were significantly altered in OSA patients with respect to controls. The fold-change analysis revealed markers of chronic kidney disease, cardiovascular risk, and oxidative stress-like indoxyl sulfate, 5-hydroxytryptamine, and 5-aminolevulenic acid, respectively, which were significantly upregulated in OSA patients.

**Conclusion:** Identifying these metabolic signatures paves the way to monitor comorbid disease progression due to OSA. Results of this study suggest that blood plasma-based biomarkers may have the potential for disease management.

## Introduction

Obstructive sleep apnea (OSA) is defined as a partial or complete obstruction of the respiratory flow through the upper airway during sleep. The severity of OSA is determined by calculating the number of episodes of apnea–hypopnea/hour during sleep and is classified based on the apnea–hypopnea index (AHI) as mild, moderate, and severe. Severe OSA is associated with more than 30 episodes per hour, whereas moderate OSA is associated with 15–30 episodes per hour. It ranges from 5–15 episodes per hour for mild OSA patients (Goyal and Johnson, 2017). The usual consequences of OSA lead to dysregulated sleep–wake cycles, changes in intrathoracic pressure, nocturnal hypoxemia, and confusional arousal (Patil et al., 2007). The prevalence of OSA is also high in obese men and women (Senaratna et al., 2017). It is believed that more than 20% of the adult population suffers from sleep-related breathing disorders, with rates as high as 50% in some countries (Heinzer et al., 2015).

Overall, the OSA population prevalence ranged from 9%–38% and is higher in men. It increased with age; the prevalence in the general adult population ranged from 6%–17% (Senaratna et al., 2017). Despite its enormous prevalence, most people remain underdiagnosed and untreated (Redline, 2017). Evidence suggested that sleep disturbance causes a wide range of physiological and psychological complications associated with chronic health issues such as chronic obstructive pulmonary disease, chronic kidney disease, and cardiovascular disorders (McNicholas, 2017; Collen et al., 2020; Lin, Lurie, and Lyons, 2020). OSA has been identified as a major health concern worldwide due to its rising prevalence, high burden of associated illness, and resulting economic impact on healthcare systems.

The gold-standard procedure to clinically diagnose OSA is overnight polysomnography (PSG). Although conventional respiratory polygraphy has helped simplify and ease the diagnosis of OSA, it is still a time- and resource-intensive endeavor. Questionnaire-based assessments are currently the most validated tools for disease development and risk screening, with the STOP-Bang questionnaire being the most accurate (Chung, Abdullah, and Liao, 2016; Chiu et al., 2017). Efforts to identify and deploy alternate ways to facilitate early and successful OSA diagnosis are under investigation. With this approach and therapeutic setting, practitioners would benefit greatly from detecting metabolic biomarkers in conveniently available bio-specimens. Despite this, there is a general lack of clinical biomarkers that can precisely detect a patient’s severity and disease pathophysiology.

Metabolomics is the new high-throughput analytic discipline that attempts to analyze in depth the full metabolome present in a biological specimen. It can help identify the novel diagnostic biomarker to understand OSA and the complex overlap syndromes. Changes in these traits, which act as molecular/metabolic fingerprints of disease development, can aid in discovering novel and promising metabolite-based biomarkers. It can also provide useful information that can aid in understanding disease pathophysiology. In the case of OSA, a heterogeneous life-threatening disease, high-throughput techniques such as metabolomics can provide a more precise status of the physiopathology of the disease. In this study, we investigated the whole plasma metabolome of patients with severe OSA using untargeted metabolomics profiling to identify diagnostic metabolic-based biomarker and elucidate potential pathophysiological mechanisms underlying the condition. We also investigated how other associated comorbidities and disease severity affected the circulating metabolomics profile.

## Materials and methods

### Chemicals and reagents

Ultrapure high-performance liquid chromatography (HPLC)-grade reagents were used in this study. Acetonitrile and methanol were obtained from Sisco Research Laboratories (India). Internal standard D-ribitol and pyridine (PX 2020) were purchased from Sigma Aldrich (St. Louis, MO, United States). Methoxylamine hydrochloride (89803) was purchased from Merck (Darmstadt, Germany). Alkane standard mixture (67444) was obtained from Supelco (Bellefonte, PA, United States ). N-methyl-N (trimethylsilyl) trifluoroamide (MSTFA) + 1% chlorotrimethylsilane (TMCS) (TS-48915) was procured from Thermo Fisher Scientific (Waltham, MA, United States).

### Sampling

A total of 3–4 ml of blood samples were collected in ethylene diamine tetraacetic acid (EDTA) vials. EDTA-plasma was obtained after the clinical assessments and diagnoses of OSA patients at the King George’s Medical University. Samples were immediately centrifuged at 2,000 x g for 10 min and stored at −80°C for further processing. The institutional ethical committee approved the study (ref. code: 107th ECM II B-Ph.D./P2**)**. Volunteers having OSA and healthy controls were identified as the subjects for the proposed study. Before enrolling in the study, all the volunteers provided written informed consent, including patients and controls.

### Study design and subject characteristics

**FIGURE 1:**
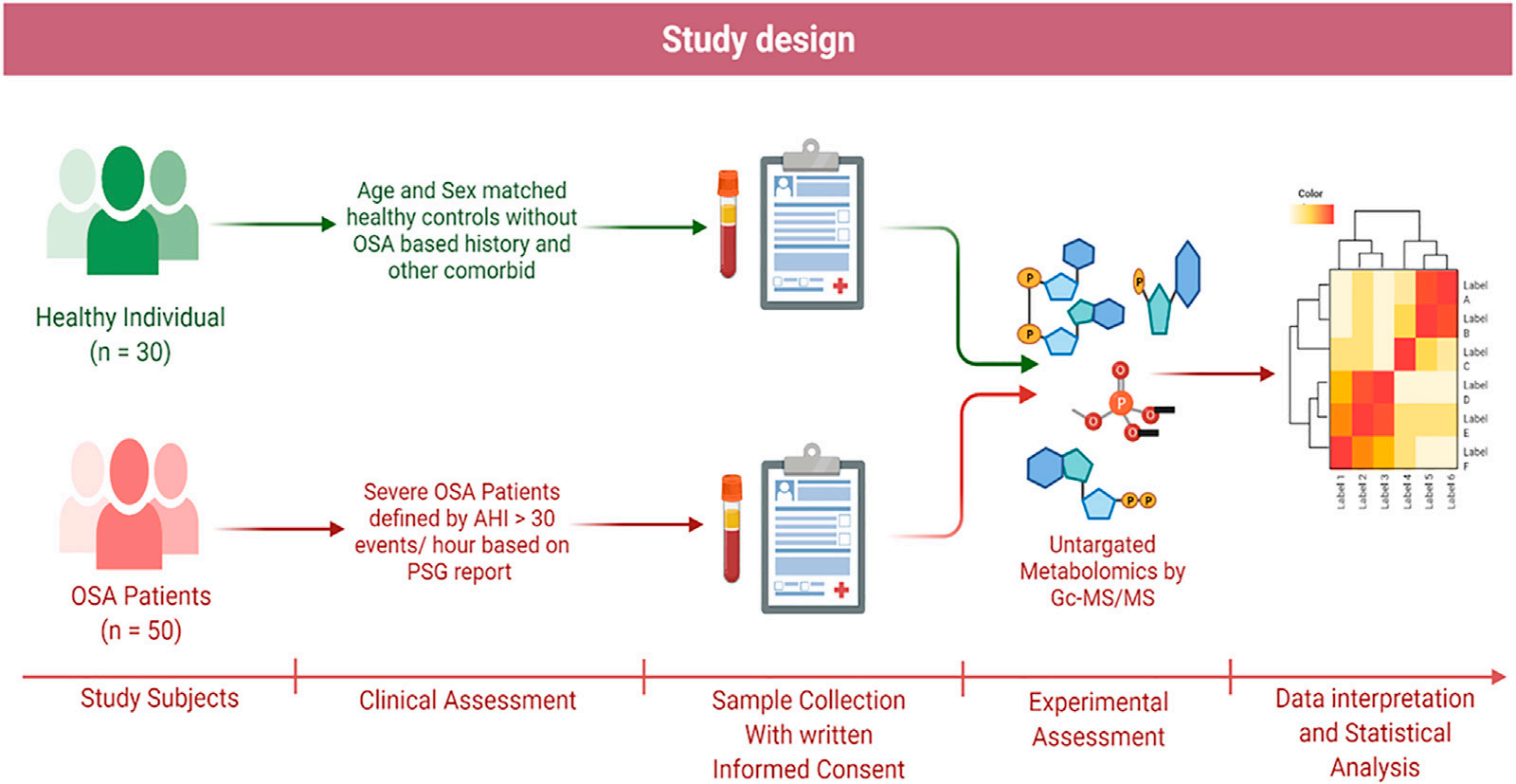
Schematic representation of study design.

The case-control study was performed by collecting plasma samples from patients referred to King George’s Medical University and the Midland Healthcare and Research Centre from March 2020 to November 2021 (Figure 1). According to the PSG reports, patients with an apnea–hypopnea index (AHI) (>30 events/hour) value exceeding the threshold were enrolled in the study. A total of 50 cases of OSA patients with a mean age of 49.74 ± 11.87 years were enrolled. Additionally, 30 controls with a mean age of 39.20 ± 3.29 years were included in the study for the comparison of metabolome profiling. This study exclusively focused on the severe OSA group without any serious comorbidity; however, few patients had hypertension. The samples were collected before CPAP therapy or any other medication was initiated. The selection criteria are based on questionnaires (for both groups) and clinical diagnosis (polysomnography of the OSA group only).

### Metabolite extraction

The metabolomic analysis was carried out on plasma samples (30 µl) following the protocol proposed by He et al. (2018) with some modifications (He et al., 2018). The plasma metabolome was extracted using 350 µl of cold methanol (−20 °C); samples were shaken using a vortex for 2 min and then centrifuged at 6,000 × g for 10 min, and the supernatant was collected and stored in a glass vial. This procedure was repeated three times, and supernatants were mixed. To the pellets, 300 µl of water (4°C) was then added, the samples were again vortexed and centrifuged as previously described, and the supernatant was mixed with the methanolic fraction. To the extracted sample, 40 µl of ribitol (0.005 mg/ml) was added as the internal standard, and the samples were dried. For derivatizing the samples, 40 μl of methoxyamine solubilized in pyridine (20 mg/ml) was added and the samples were incubated at 60^∘^C for 1 h in an orbital shaker (950 rpm). After methoximation, the samples were silylated by adding 60 μl of MSTFA+1% TMCS for 1 h at 60°C.

### Acquisition of GC-MS data

Derivatized samples were injected in the gas chromatography–mass spectrometry (GC-MS) system, and an alkane standard mixture (C10–C40 all even) was injected at the start and end of the sample analysis for retention index (RI) calculation. For the analysis of metabolites extracted from plasma samples, 1 μl of derivatized samples was injected into the splitless mode using a Triplus 100 autosampler (Thermo Scientific) in the Trace 1300 gas chromatograph equipped with a TSQ 8000 mass spectrometer. Metabolites were separated on a TraceGOLD TG-5MS column (Thermo Scientific) with a diameter of 0.25 mm, a thickness of 0.25 µm, and a length of 30 m. Ultra-high purity grade helium and argon were used as the carrier gas and collision gas, respectively, with a flow rate of 1 ml min^−1^. The injector port temperature was set at 200°C, whereas the transfer line and ion source temperature were set at 250°C. The GC program was started with an initial oven temperature of 50°C and held for 1 min; then, the temperature was increased to 100°C at a rate of 6°C min^−1^, ramped up to 200°C at 4°C min^−1^, and finally to 280 C at the rate of 20°C min^−1^ which was kept constant for 3 min. All the samples were run on full scan mode ranging from m/z 60 to 650 for the metabolite data acquisition, and raw data obtained were collected for further analysis.

### Data processing

Raw GC-MS mass spectra were converted into .abf using Abf Converter ( *http://prime.psc* *. riken. jp/Metabolomics_Software/MS-DIAL/index.html*) and processed using MS Dial 4.80 for smoothing, peak detection, peak spotting, centroiding spectra, deconvolution of the MS/MS spectrum, alignment, filtering, and annotation. For MS-DIAL data annotations, we used an in-house library built with publicly available MS spectra. For metabolite annotation and assignment of the EI-MS spectra, we followed the Metabolomics Standards Initiative (MSI) guidelines for metabolite identification (Sansone et al., 2007), using level 2 (identification based on the spectral database) and level 3 (putatively characterized compound class based on spectral similarity to known compounds of a chemical class as suggested).

Metabolomic data were analyzed using Metaboanalyst 5.0 (Chong and Xia, 2020). Internal-standard normalized datasets were transformed through “Log normalization” and scaled through Pareto scaling. Multivariate analysis was then performed to identify metabolites involved in the group’s discrimination, combining principal component analysis (PCA) plots with orthogonal partial least squares discriminant analysis (OPLS-DA) plots. Selection of features with the highest discriminatory power was based on their variable importance in projection (VIP) score >1. The variation was reproduced in the permutation test. Predictive relevance was considered when *R* ^2^ and Q^2^ values were higher than 0.5 and *p* ≤ 0.05 (Moltu et al., 2014).

A univariate response approach was used on log-transformed Pareto-scaled data in the relative concentration table to expand the results of the multivariate analyses. The Student’s t-test (*p* ≤ 0.05) was used to compare controls and OSA patient groups, followed by the application of the false discovery rate (FDR) correction for multiple comparisons to minimize false positives (*p* ≤ 0.05). A volcano plot analysis was carried out using a fold-change (FC) > 1.5 and an FDR-corrected *p*-value ≤0.05 to reduce significant feature detection by focusing on those significant metabolites with a high FC. Furthermore, to identify the metabolite coverage and the main pathways altered by OSA, data were analyzed using the enrichment analysis and the pathway analysis tool METPA (Xia and Wishart, 2011). In the pathway analysis, only pathways significantly affected (FDR-corrected *p*-value ≤0.05) and with an impact higher than 0.2 were considered affected by OSA .

### Logistic regression model analysis

Receiver operating characteristic (ROC) curve analysis was used to examine the discrimination potential of selected metabolites between OSA and controls or the diagnostic efficacy of the selected metabolite in OSA patients. Figure 6A shows an ROC curve based on the best models, and 1,000-time permutation shows the predictive accuracy and true positive rate (*p* ≤ 0.003) of the created biomarker model (Figure 6B).

## Results

### Multivariate exploratory data analysis reveals metabolic signatures in severe OSA and control groups

**FIGURE 2:**
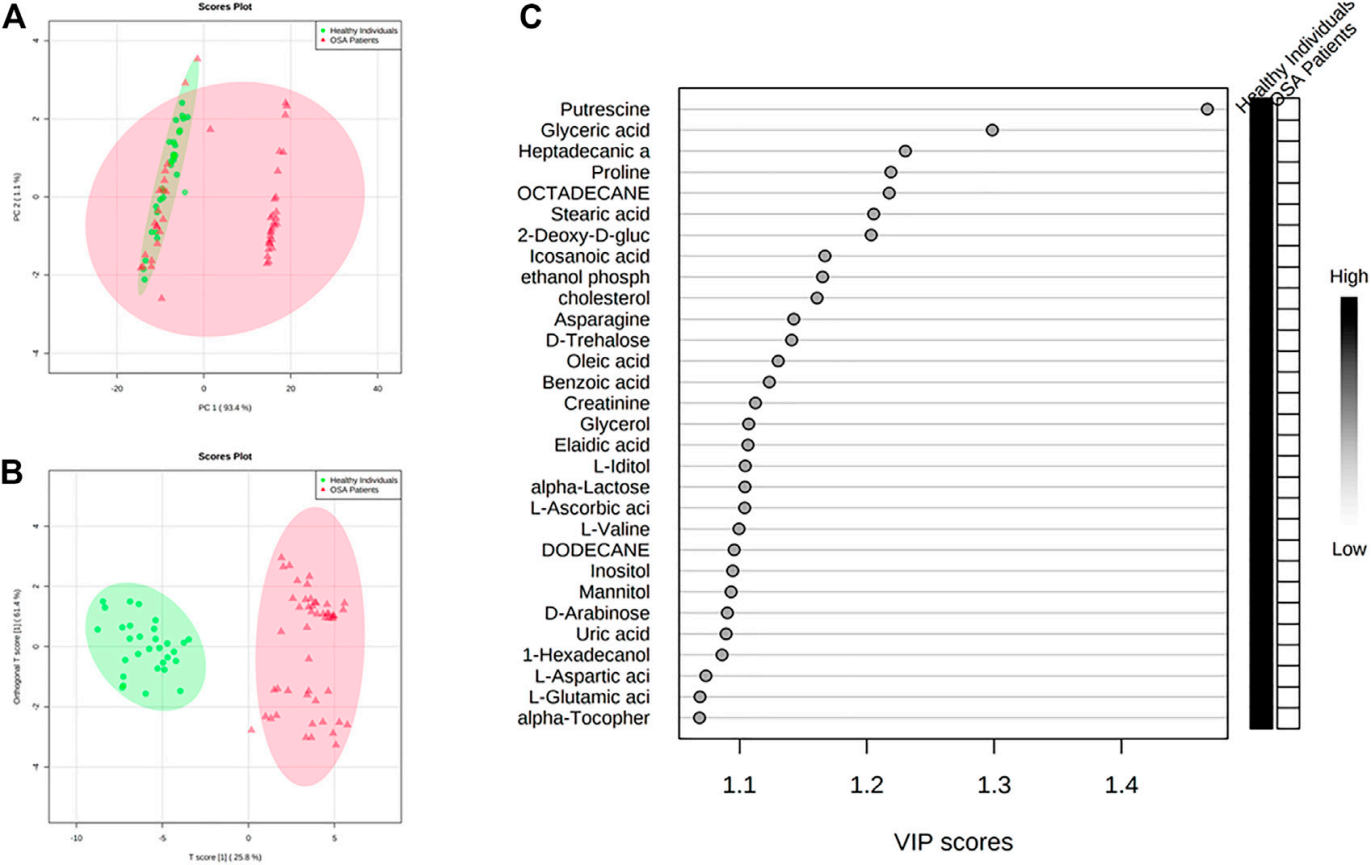
Discrimination through principal component analysis (PCA) and ortho partial least square discriminant analysis (OPLS-DA) of the metabolites patterns in controls and patients affected by OSA **(A)** PCA and **(B)** OPLS-DA plots that allowed groups discrimination by virtue of the first two components (PCs); **(C)** VIP scores of the OPLS-DA analysis.

**FIGURE 3:**
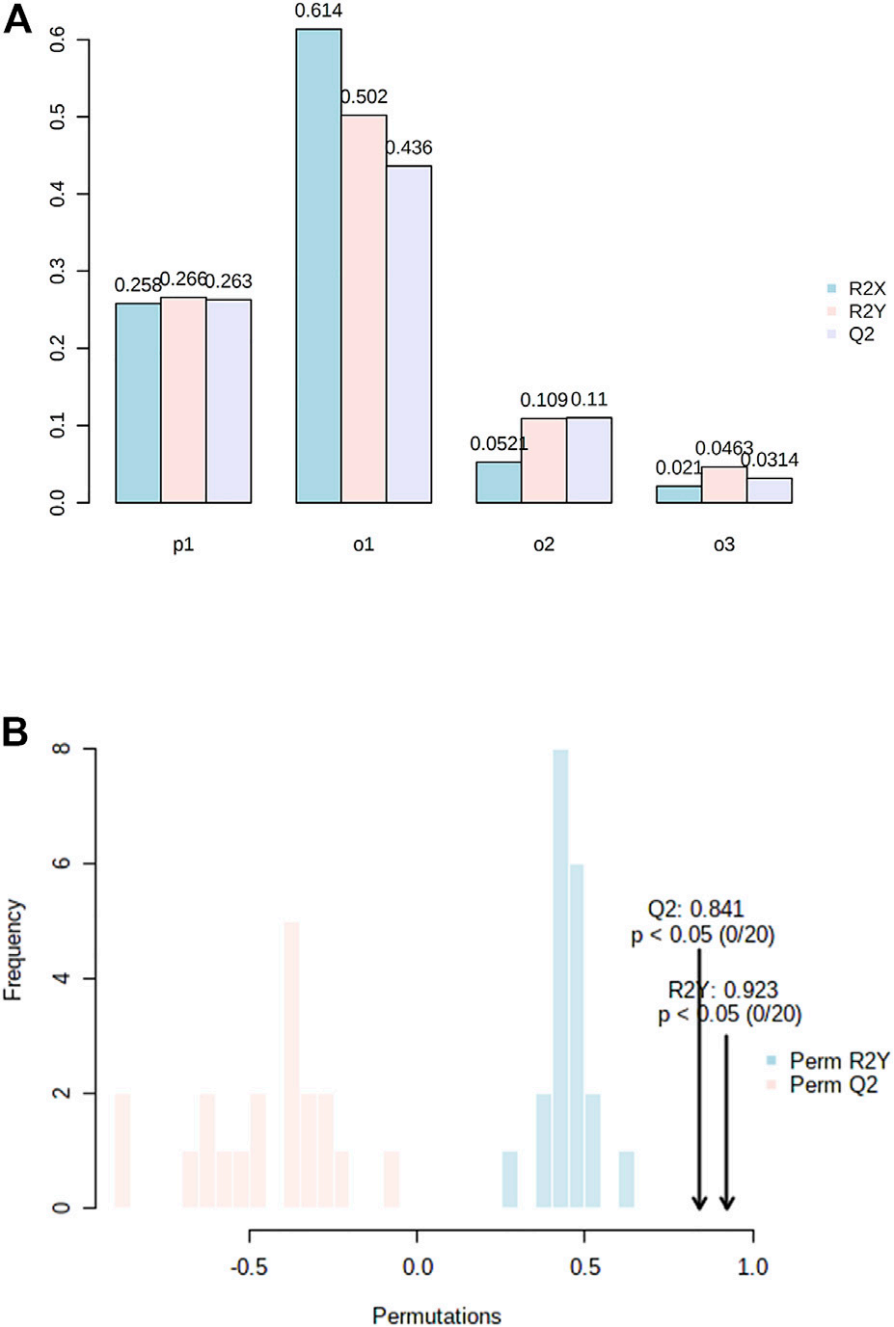
**(A)** Model overview of the OPLS-DA model for the provided dataset. It shows the R2X, R2Y, and Q2 coefficients for the groups; **(B)** Permutation analysis, showing the observed and cross-validated R2Y and Q2 coefficients.

All the patients were classified as severe OSA patients based on the AHI index (>30 events/hour) or a correlation of OSA with different parameters, including questionnaire assessment (Supplementary Data 1). The characteristics of OSA patients and controls are listed in Table 1. The analysis revealed grouped and individual metabolites that allowed sample discrimination. Among all the analyzed samples, the metabolomic analysis allowed us to relatively quantify 92 putatively annotated metabolites and extract 3,065 unknown EI-MS shared features. The unsupervised PCA, built by *virtue* of the first two components (PCs), accounted for 94.5% of the total variance. In particular, PC1 described 93.4%, whereas PC2 described 1.1% of the total variance (Figure 2A). The overlapping samples show similarities in the metabolite level among OSA and controls (22 out of 50). Therefore, to increase group separation and obtain maximal covariance between the metabolite levels, the OPLS-DA model was applied (Figure 2B). The OPLS-DA-derived loadings’ variable importance in the projection (VIP) scores revealed that more than 30 metabolites with a VIP score higher than 1 contributed to group separation, and all of them were significantly low in OSA patients (Figure 2C). In particular, putrescine, glyceric acid, heptadecanic acid, proline, octadecane, and stearic acid, among others, were the features with the highest VIP scores (Figure 2C). The model was validated to avoid overfitting through a permutation test characterized by significant (*p* ≤ 0.05) *R* ^2^ (0.841) and Q^2^ (0.923). The OPLS-DA improved group separation, pointing out separate clustering between control and OSA patients (Figures 3A,B).

**TABLE 1:** Baseline characteristics of case and controls.

Variable		Controls		Cases		*p*-value
		Mean	±SD	Mean	±SD	
Age	Years	39.20	3.29	49.74	11.87	< 0.001^*^
Height	cm	169.97	6.73	169.04	7.57	0.583
Weight	kg	73.33	11.33	90.98	14.75	< 0.001^*^
Neck size	cm	33.40	1.67	43.14	1.44	< 0.001^*^
	N	%	n	%		
Gender (n,%)	Male	30	100.0	50	100.0	-
	Female	-		-	-	
Hypertension	Yes	0	0.0	10	20.0	< 0.001^*^
	No	30	100.0	40	80.0	
BMI (> 35 kg/m^2^)	Yes	0	0.0	18	36.0	-
	No	30	100.0	32	64.0	
High risk of OSA	Yes	0	0.0	50	100.0	< 0.001^*^
	No	30	100.0	0	0.0	
EPWORTH	Would never doze	24	80.0	0	0.0	< 0.001^*^
	Slight change of dozing	6	20.0	0	0.0	
	Moderate change of dozing	0	0.0	0	0.0	
	High change of dozing	0	0.0	50	100.0	
BERLIN (high risk)	Yes	0	0.0	50	100.0	< 0.001^*^
	No	30	100.0	0	0.0	

### Univariate analysis and metabolite paneling for prediction of OSA severity and comorbidities

The univariate analysis carried out on all the annotated metabolites through the *t*-test pointed out that 91 metabolites were significantly altered in OSA patients (Table 2). Looking at the t-stat, it was possible to highlight that 12 out of 91 metabolites were significantly accumulated (negative t-stat values) in OSA patients, whereas 79 metabolites were decreased in their concentrations (positive t-stat values) (Table 2).

**TABLE 2:** Significantly different metabolites obtained through Student’s t-test (nominal *p*-value ≤ 0.05; FDR cut-off < 0.05), altered by OSA. Positive t-stat indicates down-accumulated metabolites, whereas negative t-stat indicates up-accumulated metabolites.

Metabolite name	t-stat	p-value	-log10(*p*)	FDR
Xylitol	−4.9060	4.98E-06	5.303	9.06E-06
Beta-ketoadipic acid	−4.8109	7.19E-06	5.143	1.21E-05
Indoxyl sulfate	−4.6746	1.21E-05	4.9164	1.78E-05
Tryptamine	−4.5753	1.77E-05	4.7533	2.46E-05
Diglycerol	−4.3967	3.43E-05	4.4643	4.61E-05
5-hydroxytryptamine	−4.3299	4.39E-05	4.3578	5.62E-05
Threonic acid	−3.97	0.000159	3.7992	0.000193
L-Malic acid	−3.9014	0.000202	3.6957	0.000241
Myristic acid	−3.3816	0.001129	2.9474	0.001253
5-Aminolevulinic acid	−3.0052	0.003568	2.4475	0.003648
Methyltetrahydrophenanthrenone	−2.7417	0.007577	2.1205	0.007661
DL-Isocitric acid	−2.2315	0.02852	1.5448	0.02852
Pyroglutamic acid	3.0179	0.003437	2.4638	0.003554
1_5-Anhydro glucitol	3.0749	0.002902	2.5373	0.003035
Glucose	3.0760	0.002893	2.5387	0.003035
Leucine	3.1537	0.002289	2.6403	0.002451
Hexaric acid	3.1979	0.002001	2.6988	0.002167
Meso-erythritol	3.3020	0.00145	2.8387	0.00159
Lysine	3.5545	0.000647	3.1891	0.000727
Glucose-1-phosphate	3.7512	0.000337	3.473	0.000383
Palmitic acid	3.8664	0.000227	3.6434	0.000262
Glycine	3.8842	0.000214	3.6699	0.000249
Succinic acid	3.8846	0.000213	3.6706	0.000249
Citric acid	4.0874	0.000105	3.9784	0.000129
D-fructose	4.1308	9.01E-05	4.0454	0.000112
Glutamic acid	4.2437	6.00E-05	4.2216	7.59E-05
Cadaverine	4.3617	3.90E-05	4.4085	5.08E-05
D-galactosamine	4.3644	3.87E-05	4.4127	5.08E-05
Tyrosine	4.3959	3.44E-05	4.463	4.61E-05
Tryptophan	4.5722	1.79E-05	4.7482	2.46E-05
Maltose	4.5724	1.78E-05	4.7485	2.46E-05
Indole-3-acetic acid	4.5838	1.71E-05	4.7672	2.46E-05
Dopamine	4.6748	1.21E-05	4.9168	1.78E-05
L-norvaline	4.6788	1.19E-05	4.9234	1.78E-05
Hexadecanoic acid	4.7221	1.01E-05	4.9951	1.56E-05
1_4-Benzenedicarboxylic acid	4.7701	8.42E-06	5.0749	1.32E-05
Lactitol	4.7717	8.36E-06	5.0776	1.32E-05
Lauric acid	4.7929	7.71E-06	5.113	1.25E-05
Ornithine	4.8053	7.35E-06	5.1336	1.22E-05
Threonine	4.8117	7.17E-06	5.1444	1.21E-05
Ethanolamine	4.8631	5.88E-06	5.2308	1.03E-05
Alanine	4.8983	5.13E-06	5.2901	9.15E-06
N-acetyl-d-glucosamine	4.9239	4.64E-06	5.3332	8.62E-06
Phenylalanine	4.9323	4.49E-06	5.3476	8.52E-06
2-Aminoethanol	4.9348	4.45E-06	5.3519	8.52E-06
L-serine	4.9353	4.44E-06	5.3526	8.52E-06
Methionine	5.0035	3.40E-06	5.4686	6.87E-06
Uridine	5.0048	3.38E-06	5.4708	6.87E-06
L-cystine	5.0682	2.64E-06	5.5791	5.58E-06
Melibiose	5.0771	2.54E-06	5.5945	5.51E-06
Tyramine	5.0827	2.49E-06	5.604	5.51E-06
Glucuronate	5.1141	2.20E-06	5.6581	5.00E-06
Hydroxyproline	5.1464	1.93E-06	5.7137	4.51E-06
Pipecolic acid	5.215	1.47E-06	5.8325	3.52E-06
D-arabinose	5.2574	1.24E-06	5.9062	3.05E-06
Isoleucine_major	5.3024	1.04E-06	5.9848	2.62E-06
Gluconic acid	5.3346	9.10E-07	6.041	2.37E-06
Mannitol	5.3588	8.25E-07	6.0835	2.21E-06
Iminodiacetate	5.379	7.60E-07	6.119	2.10E-06
L-glutamic acid	5.3809	7.55E-07	6.1223	2.10E-06
Ribose	5.3902	7.27E-07	6.1387	2.10E-06
Alpha-tocopherol	5.3906	7.26E-07	6.1393	2.10E-06
1-Hexadecanol	5.4139	6.60E-07	6.1804	2.07E-06
Myo-inositol	5.4149	6.57E-07	6.1822	2.07E-06
Norleucine	5.4274	6.25E-07	6.2043	2.07E-06
Inositol	5.4758	5.13E-07	6.2898	1.80E-06
Pentadecanoic acid	5.4765	5.12E-07	6.2911	1.80E-06
L-aspartic acid	5.4877	4.89E-07	6.3109	1.80E-06
Alpha-lactose	5.527	4.16E-07	6.3806	1.65E-06
Dodecane	5.5303	4.11E-07	6.3865	1.65E-06
Uric acid	5.5543	3.72E-07	6.4292	1.61E-06
L-valine	5.6082	2.98E-07	6.5253	1.36E-06
L-iditol	5.6147	2.90E-07	6.5369	1.36E-06
Elaidic acid	5.6284	2.74E-07	6.5615	1.36E-06
Benzoic acid	5.6409	2.61E-07	6.5838	1.36E-06
Glycerol	5.6857	2.17E-07	6.6642	1.23E-06
L-ascorbic acid	5.6988	2.05E-07	6.6876	1.23E-06
Creatinine	5.7135	1.93E-07	6.7141	1.23E-06
Oleic acid	5.9324	7.75E-08	7.1104	5.43E-07
D-trehalose	5.9751	6.48E-08	7.1884	4.91E-07
Asparagine	5.9801	6.35E-08	7.1976	4.91E-07
Cholesterol	5.9942	5.98E-08	7.2233	4.91E-07
Ethanol phosphate	6.1663	2.89E-08	7.5393	2.92E-07
2-Deoxy-d-glucose	6.183	2.69E-08	7.5702	2.92E-07
Icosanoic acid	6.2759	1.81E-08	7.7419	2.36E-07
Proline	6.3523	1.31E-08	7.8839	1.98E-07
Stearic acid	6.3935	1.10E-08	7.9605	1.98E-07
Octadecane	6.564	5.25E-09	8.2794	1.20E-07
Heptadecanic acid	6.6815	3.16E-09	8.5004	9.58E-08
Glyceric acid	7.4731	9.80E-11	10.009	4.46E-09
Putrescine	8.7257	3.68E-13	12.435	3.35E-11

**FIGURE 4:**
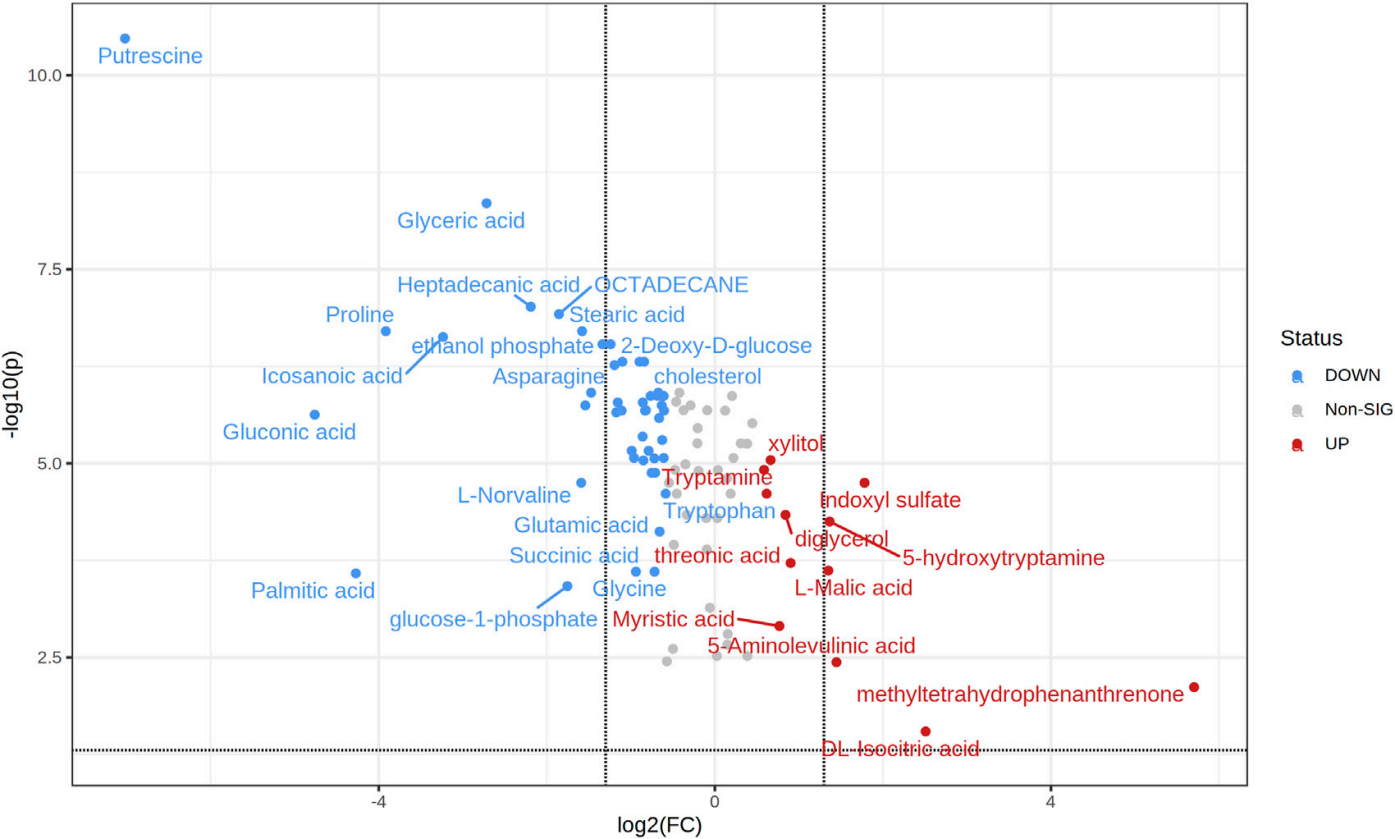
Important features selected by volcano plot with fold change threshold (x) 1.5 and FDR corrected t-tests threshold (y) P ≤ 0.05. The red (up-accumulated) and blue (down-accumulated) circles represent features above the threshold. Note both fold changes and p values are log transformed. The further its position away from the (0,0), the more significant the feature is. (N = 50 OSA and 30 = Controls).

Data were further analyzed through the volcano plot using an FC of 1.5, and an FDR-corrected *p*-value ≤ 0.05 to reduce the number of significant metabolites, restricting the attention only to those characterized by a high difference between the two groups. The analysis allowed the identification of 58 significant metabolites out of 91. In particular, 12 were up-accumulating whereas 46 were down-accumulating in OSA patients (Figure 4).

Among them, 5-hydroxytryptamine (5-HT), indoxyl sulfate, 5-aminolevulinic acid (5-ALA), DL-isocitric acid, and malic acid were present in significantly higher concentrations than the control. Indoxyl sulfate is a tryptophan derivative produced through intestinal microbiota, and its accumulation increases cellular fibrosis and oxidative stress (Lu et al., 2021). It was evident that higher levels of indoxyl sulfate represent a slowdown of the glomerular filtration rate, increased risk of chronic kidney disease, and adverse cardiovascular events (Ellis et al., 2016). Another significantly altered metabolite was 5-ALA, which has an indirect association with cellular energy generation, which might have various impacts ranging from cellular to endocrine to neurologic to behavioral (Perez et al., 2013). Furthermore, 5-ALA has been shown to affect tryptophan and serotonin levels (Daya et al., 1989). In this study, we also identified a higher level of 5-hydroxytryptamine reported as a possible target of OSA in retrospective studies (Jagannathan et al., 2017; Maierean et al., 2021). Serotonin receptors are also present in central respiratory neuronal groups, with 5-HT (1A) (inhibitory) and 5-HT (2) receptors being the most common. Stimulation of the 5-HT (2A), 5-HT (2C), and 5-HT (3) receptor subtypes in the periphery inhibit respiration by acting on the nodose ganglion. The clinical impact of 5-HT (2A) and 5-HT (3) antagonists on OSA is now being studied in trials (Veasey, 2003).

Interestingly, some important metabolites have lower accumulation, such as tryptophan, glutamic acid, glycine, proline, asparagine, and norvaline, in association with OSA. It is well-established that the amino acid metabolism is significantly altered in OSA patients (Humer, Pieh, and Brandmayr, 2020). Previously, it was hypothesized that the alteration in tryptophan metabolism might play an important role in cardiovascular comorbidities and intermittent hypoxia in OSA patients (Boulet et al., 2015). Additionally, altered levels of glutamic acid, glycine, proline, asparagine, and norvaline have also been reported in OSA patients (Lebkuchen et al., 2018; Kiens et al., 2021). Therefore, the metabolic pattern of altered metabolites may be used for better performance in the screening of OSA and propose an OSA biomarker panel.

### Enrichment and pathway analysis correlated with OSA severity and comorbidities

**FIGURE 5:**
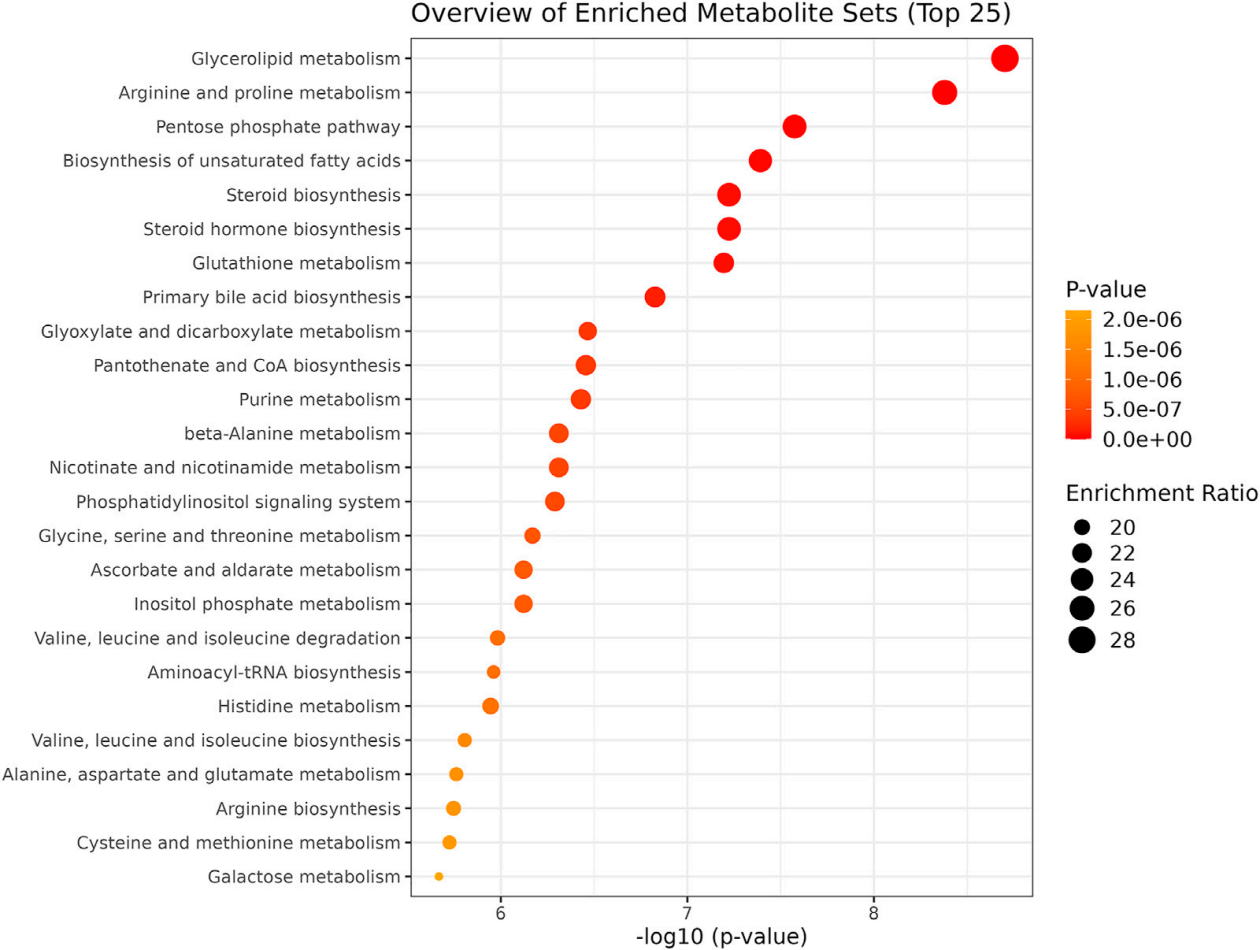
Summary plot of the Quantitative Enrichment Analysis (QEA). Enrichment ratio: the dimension of the bubble indicates the enrichment level.

We performed an enrichment analysis (Figure 5) and pathway analysis (Table 3) based on the Kyoto Encyclopedia of Genes and Genomes (KEGG) to determine which metabolic pathways were affected. When OSA plasma metabolomics data were compared with control plasma metabolomics data, 22 enriched metabolic pathways with impact scores >0.05 were identified (Figure 5). They belong to a wide range of metabolic pathways, including carbohydrate metabolism (tri-carboxylic acid cycle, starch and sucrose metabolism, galactose metabolism, and glyoxylate and dicarboxylate metabolism), amino acid metabolism (phenylalanine, tyrosine glutamine, glycine, serine, threonine, alanine, aspartate, phenylalanine tyrosine, tryptophan, arginine, proline, glutamate, cysteine, methionine, and tryptophan biosynthesis), metabolism of cofactors and vitamins (ascorbate and aldarate metabolism), lipid metabolism (glycerolipid), and other metabolism (pentose and glucuronate interconversions, glutathione, amino sugar and nucleotide sugar metabolism, and inositol phosphate metabolism) (Table 3).

**TABLE 3:** Result from the “pathway analysis” (topology + enrichment analysis) carried out on the metabolites identified in the blood of control and OSA patients.

Pathways	Total cmpd	Hits	Raw p	FDR	Impact
Phenylalanine tyrosine and tryptophan biosynthesis	4	2	9.93E-06	1.37E-05	1
D-glutamine and D-glutamate metabolism	6	1	6.00E-05	6.98E-05	0.5
Ascorbate and aldarate metabolism	8	2	7.55E-07	2.10E-06	0.5
Glycine serine and threonine metabolism	33	5	6.77E-07	2.10E-06	0.48704
Arginine and proline metabolism	38	5	4.18E-09	1.04E-07	0.4441
Alanine aspartate and glutamate metabolism	28	6	1.73E-06	3.59E-06	0.42068
Phenylalanine metabolism	10	2	9.93E-06	1.37E-05	0.35714
Glycerolipid metabolism	16	2	1.99E-09	9.93E-08	0.33022
Pentose and glucuronate interconversions	18	3	6.80E-06	1.06E-05	0.29688
Tyrosine metabolism	42	3	6.55E-06	1.06E-05	0.29407
Tryptophan metabolism	41	4	1.39E-05	1.83E-05	0.28789
Glyoxylate and dicarboxylate metabolism	32	6	9.16E-07	2.41E-06	0.25927
Arginine biosynthesis	14	3	1.79E-06	3.59E-06	0.17766
Citrate cycle (TCA cycle)	20	3	0.0001042	0.00011578	0.16723
Aminoacyl-tRNA biosynthesis	48	15	1.09E-06	2.58E-06	0.16667
Starch and sucrose metabolism	18	2	5.78E-07	1.98E-06	0.13486
Amino sugar and nucleotide sugar metabolism	37	3	1.84E-05	2.35E-05	0.13466
Inositol phosphate metabolism	30	2	7.55E-07	2.10E-06	0.12939
Cysteine and methionine metabolism	33	3	1.88E-06	3.62E-06	0.1263
Glutathione metabolism	28	6	6.38E-08	4.56E-07	0.12267
Galactose metabolism	27	4	5.95E-07	1.98E-06	0.12027

Total Compound: the total number of compounds in the pathway; Hits: the matched number from the uploaded data; Raw p: the original *p*-value, FDR: the false discovery rate applied to the nominal *p*-values to control for false-positive findings; Impact: the pathway impact value calculated from pathway topology analysis. Only pathways with an impact higher than 0.05 were reported.

### Identification of metabolite-based potential biomarkers for OSA

**FIGURE 6:**
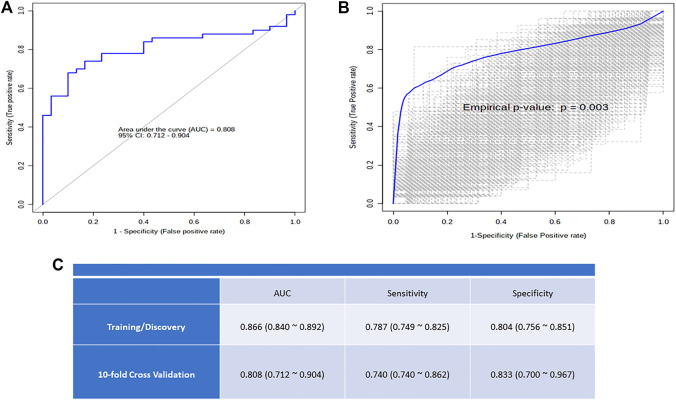
**(A)** The logistic regression ROC analysis for controls versus OSA and distribution of metabolite concentration used for model building; **(B)** Box plot of the predictive accuracy of the created biomarker model; **(C)** The plot shows the AUC of all permutations, highlighting the actual observed AUC in blue, along with showing the empirical p-value.

The onset of OSA has been known to predispose individuals to comorbidities and metabolic disorders (Gleeson and McNicholas, 2022). We used log regression analysis with Metaboanalyst to validate the efficacy of our highlighted metabolites as metabolic biomarkers in severe OSA patients compared with controls. Using a 10-fold cross validation, indoxyl sulfate 5-hydroxytryptamine, 5-aminolevulinic acid, DL-isocitric acid, and malic acid, the ROC of the study model shows an area under curve (AUC) of 0.808, sensitivity of 0.740, and specificity of 0.833. Figure 6 shows the potential of these metabolites to discriminate two groups and may act as a biomarker for OSA severity and progression.

## Discussion

For the first time, this study presents the snapshot of comparative metabolic changes that occur in patients diagnosed with severe OSA AHI (>30 events/hour) and the control group. The relative number of altered metabolites in patients with higher AHI represents the dysregulated functional endpoint of otherwise normal metabolic pathways. The frequent episodes of apnea/hypopnea are pronounced to develop intermittent hypoxemia, which causes systemic inflammatory responses, consequently accelerating disease complications (Lévy et al., 2015; Maspero et al., 2015). Inflammation has been noted as a critical factor in the pathophysiology of OSA and its complications and comorbidities (Maspero et al., 2015; Kimura et al., 2019). Anatomical predispositions make the subject prone to OSA (Mohit and Chand, 2021). However, the onset of OSA occurs at an advanced stage with comorbidities and metabolic syndromes. Meanwhile, metabolomics is a promising approach in the field of personalized medicine and care. Metabolites are closely linked to pathological disorders because they represent the essential metabolic activity and status of a biological system (DeBerardinis and Thompson, 2012). There is no defined molecular and metabolomics basis for OSA, and being a heterogeneous chronic disorder, it is very difficult to pinpoint a specific biomarker reliably. Using spectrometry-based metabolomics and untargeted analysis, we detected many metabolites (amino acids, fatty acids, and carbohydrates) that correlate with the transitional changes for normal to OSA subjects. Furthermore, based on these altered metabolites, we constructed a model of altered metabolite pathways that encompass the pathophysiology of OSA.

Our study exclusively focused on a group of severe OSA patients (AHI >30 events/hour) and their metabolic changes; compared to controls, these traits are strongly correlated with associated comorbidities. Remarkably, we identified a total of 92 metabolites which are differently abundant in both groups. Indeed, after the fold-change induction analysis, we identified the top 23 altered metabolites independently linked with several known comorbid factors. The bookmarks of this untargeted metabolomics approach mainly comprise amino acid, sugar, lipids, and their derivatives. Particularly, the pathway analysis revealed that the alteration in these molecules is strongly associated with several interlinked pathways. Obesity is the most common factor in OSA patients (Jehan et al., 2017). Collectively, in the pathway enrichment analysis, we identified the top 25 pathways that are most affected; glycerolipid metabolism and biosynthesis of fatty acids are found to be associated with obese and severe OSA patients. Additionally, amino acid metabolism and biosynthesis are remarkably affected in OSA patients.

We found that the indoxyl sulfate level was higher in patients with OSA than in the controls. The repetitive episodes of apnea/hypopnea in OSA patients increase the level of glucocorticoids and inflammatory cytokines, resulting in increased oxidative stress (Stanek, Brożyna-Tkaczyk, and Myśliński, 2021; Wang et al., 2021). Consequently, oxidative stress and redox imbalance lead to an accumulation of reactive oxygen species (ROS). It is well-reported that indoxyl sulfate induces oxidative stress by modifying the balance between pro- and antioxidant mechanisms in endothelial cells (Dou et al., 2007). Indoxyl sulfate has been documented to exhibit nephrotoxicity and is associated with the accelerated progression of chronic kidney disease (CKD) (Dou et al., 2007). Quantifying the plasma indoxyl sulfate level may have great meaning in clinical research and applications for the risk assessment of CKD in OSA patients. Indoxyl sulfate is also known to be a predictive marker for cardiovascular risk in CKD patients (Fan et al., 2019). Understanding the pathophysiology of cardiovascular risk and CKD in OSA patients might help develop new treatment strategies and interventions to reduce its morbidity and mortality.

Neurobehavioral disorders are significantly associated with OSA, such as loss of memory, lack of concentration, and poor attention span. This may even result in an increased risk of road accidents (Day et al., 1999). We also detected an increased level of serotonin (5-hydroxytryptamine) in severe OSA patients. Serotonin is a neurochemical that is actively involved in sleep modulation. However, the role of serotonin in sleep modulation is still very controversial. Preliminary studies suggested that serotonin is essential to acquire and maintain behavioral sleep (permissive role on sleep) (Portas, Bjorvatn, and Ursin, 2000). Serotonin has been linked to fatigue because of its well-known effects on sleep, drowsiness, and loss of concentration; these symptoms are experienced by most OSA patients (Meeusen et al., 2006; Lal et al., 2021). According to the revised central fatigue hypothesis, we suggested that an increased level of serotonin is associated with feelings of tiredness and accelerates the onset of fatigue in severe OSA patients. At the same time, a low serotonin level favors improving sleep quality (Meeusen et al., 2006).

In our study, we found some important amino acids that are significantly altered in severe OSA patients, such as tryptophan, glycine, and proline. Tryptophan metabolism is a key regulator of several pathways associated with intermittent hypoxia in OSA patients (Meeusen et al., 2006). Recent studies have suggested that an alteration in tryptophan metabolism may play an important role in comorbidities related to cardiovascular risk and might be interlinked with cancer progression in OSA (Boulet et al., 2015). On the other hand, our study suggested that reduced amino acid levels in OSA patients may indicate that this metabolic disorder appears to be associated with the acceleration of nucleic acid biosynthesis in OSA patients. Increased levels of 5-aminolevulinate were significantly identified in severe OSA patients. Alteration in 5-aminolevulinate has been shown to reduce the saturation of tryptophan and serotonin in the brain. Tryptophan is also known to be a precursor of melatonin; thus, the alterations in tryptophan metabolism have been associated with depressive disorders in humans (Daya et al., 1989).

In this study, we also identified that several intermediate metabolites associated with the TCA cycle, such as isocitrate and malate, were significantly increased in the plasma sample of severe OSA patients. The body’s energy is mainly obtained from the TCA cycle (Lima, Martins-Santos, and Chaves, 2015). It is hypothesized that the accumulation of these intermediate metabolites is significantly associated with energy metabolism. In addition, branched-chain amino acids are another source of energy which are also altered and associated with energy demand in OSA patients. This study also suggested that the alterations in plasma amino acids, fatty acids, and intermediate TCA cycle metabolites are directly or indirectly associated with energy production to meet the growing demand for energy in OSA patients, especially in severe cases. Together, these untargeted metabolomics results are aligned and show that the observed metabolite changes could indicate OSA progression and impairment in CKD and other comorbidities.

Some limitations are worth noting in this study. Considering the infancy of this field, an exploratory approach was adopted to gain new insights from which new hypotheses could be developed. We need further development of a confirmatory targeted analysis to validate our findings to determine the absolute concentration (only relative concentrations were used in this work) of metabolites in other independently selected groups. Another potential limitation is that the sample taken from control subjects was young, with a lower BMI and without hypertension. However, these differences are because OSA is more frequent in patients with higher BMI and older age. In addition, an orthogonal technique such as liquid chromatography–mass spectrometry (LC-MS) with more comprehensive metabolic coverage and less complex sample preparation steps (i.e., drying and derivatization) may have been helpful in the identification and relative quantification of a higher number of metabolites belonging to more pathways or increasing the coverage of the pathways already identified to be involved in OSA responses.

## Conclusion

In this GC/MS-based untargeted metabolomics study, we found out the metabolite-based biomarker concerning the severity of OSA. This novel “omics” strategy and analytical analysis will be helpful in exploring the associated role of metabolite alteration in a population at a great risk of OSA. As several metabolisms and pathway-based mechanisms of OSA have been put forward, the exact mechanism of translational relevance remains elusive. The identified metabolites are mainly the group of amino acids, lipids, and carbohydrates, which might be associated with disease severity and comorbidities, when compared with the control group. Additionally, we identified several metabolites for the first time in severe OSA patients. The increased level of these metabolites can predispose OSA patients to oxidative stress resulting in tissue injury, cardiovascular disorders, and CKD. This study is framed to understand the theoretical basis of metabolomics and molecular factors; information would be beneficial in exploring therapeutic intervention possibilities.

## Data Availability

The raw data supporting the conclusion of this article will be made available by the authors, without undue reservation.
