# Supplementary material for: Identification of metabolic fingerprints in severe obstructive sleep apnea using gas chromatography–Mass spectrometry
Source: Front Mol Biosci. 2022 Nov 21;9:1026848. doi: 10.3389/fmolb.2022.1026848 (PMC9732946; doi:10.3389/fmolb.2022.1026848)
Supplement: Supplementary file 2 [file Table1.DOCX]

**Supplementary Table – 1: Correlations of OSA Parameters with Age, Physical Status and Questionnaire-based assessment.**

| Pearson Correlation | AHI | | STOP BANG | | BERLIN | |
| --- | --- | --- | --- | --- | --- | --- |
|  | r-value | p-value | r-value | p-value | r-value | p-value |
| Age | -.239 | .095 | -.023 | .874 | -.025 | .865 |
| Height in cm | .018 | .900 | .111 | .443 | .085 | .556 |
| Weight in kg | .351 | **.013** | .379 | **.007** | .350 | **.013** |
| Neck Size in cm | .471 | **.001** | .447 | **.001** | .458 | **.001** |

**Supplementary Table – 2: Mutual Correlations between OSA Parameters**

| Pearson Correlation | AHI | | STOP BANG | |
| --- | --- | --- | --- | --- |
|  | r-value | p-value | r-value | p-value |
| STOP BANG | .648 | **<0.001** | - | - |
| BERLIN | .647 | **<0.001** | .636 | **<0.001** |

**Supplementary Table – 3: Comparison of AHI Score with Obesity and Hypertension**

| Group | | AHI | | Unpaired t test | |
| --- | --- | --- | --- | --- | --- |
|  |  | Mean | SD | t-value | p-value |
| Obesity | Non-Obese | 61.84 | 21.93 | 2.01 | 0.050 |
|  | Obese | 79.34 | 34.08 |  |  |
| Hypertension | No Hypertension | 59.24 | 20.75 | 2.37 | **0.022** |
|  | Hypertension | 76.19 | 29.70 |  |  |

**Supplementary Table – 4: Comparison of Stop Bang Score with Obesity and Hypertension**

| Group | | STOP BANG | | Unpaired t test | |
| --- | --- | --- | --- | --- | --- |
|  |  | Mean | SD | t-value | p-value |
| Obesity | Non-Obese | 7.10 | 0.71 | 1.60 | 0.117 |
|  | Obese | 7.50 | 0.71 |  |  |
| Hypertension | No Hypertension | 7.00 | 0.72 | 2.48 | **0.017** |
|  | Hypertension | 7.50 | 0.62 |  |  |

**Supplementary Table – 5: Comparison of Berlin Score with Obesity and Hypertension**

| Group | | BERLIN | | Unpaired t test | |
| --- | --- | --- | --- | --- | --- |
|  |  | Mean | SD | t-value | p-value |
| Obesity | Non-Obese | 2.43 | 0.50 | 2.85 | **0.006** |
|  | Obese | 2.90 | 0.32 |  |  |
| Hypertension | No Hypertension | 2.44 | 0.50 | 1.56 | 0.124 |
|  | Hypertension | 2.67 | 0.49 |  |  |
